# DeepPFP: a multi-task-aware architecture for protein function prediction

**DOI:** 10.1093/bib/bbae579

**Published:** 2025-02-05

**Authors:** Han Wang, Zilin Ren, Jinghong Sun, Yongbing Chen, Xiaochen Bo, JiGuo Xue, Jingyang Gao, Ming Ni

**Affiliations:** College of Information Science and Technology, Beijing University of Chemical Technology, No. 15 North Third Ring East Road, Chaoyang District, Beijing 100029, China; Changchun Veterinary Research Institute, Chinese Academy of Agricultural Sciences, State Key Laboratory of Pathogen and Biosecurity, Key Laboratory of Jilin Province for Zoonosis Prevention and Control, Changchun 130122, China; School of Information Science and Technology, Northeast Normal University, Changchun 130117, China; College of Information Science and Technology, Beijing University of Chemical Technology, No. 15 North Third Ring East Road, Chaoyang District, Beijing 100029, China; Changchun Veterinary Research Institute, Chinese Academy of Agricultural Sciences, State Key Laboratory of Pathogen and Biosecurity, Key Laboratory of Jilin Province for Zoonosis Prevention and Control, Changchun 130122, China; School of Information Science and Technology, Northeast Normal University, Changchun 130117, China; Advanced & Interdisciplinary Biotechnology, Academy of Military Medical Sciences, No. 27 Taiping Road, Haidian District, Beijing 100850, China; Advanced & Interdisciplinary Biotechnology, Academy of Military Medical Sciences, No. 27 Taiping Road, Haidian District, Beijing 100850, China; College of Information Science and Technology, Beijing University of Chemical Technology, No. 15 North Third Ring East Road, Chaoyang District, Beijing 100029, China; Advanced & Interdisciplinary Biotechnology, Academy of Military Medical Sciences, No. 27 Taiping Road, Haidian District, Beijing 100850, China

**Keywords:** protein function prediction, SARS-CoV-2, deep learning, meta learning

## Abstract

Deriving protein function from protein sequences poses a significant challenge due to the intricate relationship between sequence and function. Deep learning has made remarkable strides in predicting sequence-function relationships. However, models tailored for specific tasks or protein types encounter difficulties when using transfer learning across domains. This is attributed to the fact that protein function relies heavily on structural characteristics rather than mere sequence information. Consequently, there is a pressing need for a model capable of capturing shared features among diverse sequence-function mapping tasks to address the generalization issue. In this study, we explore the potential of Model-Agnostic Meta-Learning combined with a protein language model called Evolutionary Scale Modeling to tackle this challenge. Our approach involves training the architecture on five out-domain deep mutational scanning (DMS) datasets and evaluating its performance across four key dimensions. Our findings demonstrate that the proposed architecture exhibits satisfactory performance in terms of generalization and employs an effective few-shot learning strategy. To explain further, Compared to the best results, the Pearson’s correlation coefficient (PCC) in the final stage increased by ~0.31%. Furthermore, we leverage the trained architecture to predict binding affinity scores of the DMS dataset of SARS-CoV-2 using transfer learning. Notably, training on a subset of the Ube4b dataset with 500 samples resulted in a notable improvement of 0.11 in the PCC. These results underscore the potential of our conceptual architecture as a promising methodology for multi-task protein function prediction.

## Introduction

The functions and properties of proteins, along with protein–protein interactions, are fundamental to numerous biological processes [1]. Mapping sequence to function presents a critical challenge for protein optimization and rational design, particularly because sequence space expands exponentially with length, and traditional wet-lab methods cannot comprehensively characterize most proteins’ functions [2]. Additionally, the limited data from wet experiments and the long-tailed distribution of labels complicate the application of traditional statistical learning and machine learning methods to sequence-function mapping. Long-tail distribution describes the uneven distribution of labels in the data, characterized by a large proportion of individuals with low values. This phenomenon makes it more difficult for the model to learn the distribution of rare individuals, leading to a bias against rare but potentially high functional score individuals, thereby making it difficult to generalize to rare individuals. Deep mutational scanning (DMS) techniques generate libraries of protein variants that exhibit high sequence similarity, characterized by only a few amino acid substitutions. This substantial similarity poses significant challenges for computational methods in handling the sensitivity to point mutations [3]. The characteristics of DMS require computational models to be sensitive enough to detect subtle changes that may have important biological impacts. However, predictive models may learn these similarities instead of the mutations that actually have a significant impact on function, thereby ignoring key biological features that may only be present in a few sequences.

In recent years, deep learning has emerged as a promising approach to address sequence-function mapping problems. This data-driven technology relies on substantial datasets to learn the protein variant sequence space. Theoretically, it is possible to design a suitable model to fit a specific task with enough data [4–7]. However, neural networks (NNs) often lack the capability for human-like systematic generalization, meaning they struggle to formulate novel combinations from known knowledge. This limitation significantly impairs deep learning models’ predictive performance when data is scarce or when generalized to out-of-domain data [8]. Owing to the similarity between human languages and proteins, language models have been progressively adapted into protein language models (PLMs). PLMs leverage advanced sequence modeling capabilities to overcome the data scarcity challenge by better generalizing from the available sequences to unseen protein variants. However, they introduce new challenges such as the need for significant computational resources and the risk of overfitting on specific protein families without sufficient regularization. Several PLMs based on unsupervised learning, including EVmutation [9] and Evolutionary Scale Modeling (ESM) [10–13] have demonstrated the ability to learn and capture protein structural and functional representations from different protein data. However, for specific downstream tasks such as DMS, retraining the decoder is necessary, with limited adaptability across multiple tasks. Due to limited training data, PLMs are poorly scalable when applied to different tasks. Meta-learning emerges as a potent solution here by enabling models to rapidly adapt to new tasks. This ‘learn to learn’ approach addresses both the challenge of domain drift in traditional transfer learning and enhances the applicability of decoders across varied protein analysis tasks by leveraging prior learned knowledge to make more informed predictions on new, related tasks [14–17]. In other words, PLMs can effectively representation of protein sequences, and decoders trained with meta-learning can quickly adapt to new tasks. Meta-learning applications span various fields, including medical image analysis [18], drug discovery [19], drug response prediction [2, 0], and antigen–antibody binding recognition [21]. For instance, Chen et al. [19] utilized meta-learning combined with graph attention networks to capture molecular-level interactions, thereby demonstrating its capability for few-shot tasks. Liu et al. [18] proposed a multiple learner based on Few-shot learning (FSL) for medical image classification, achieving state-of-the-art performance. Liu et al. [20] developed Pan-Peptide Meta-Learning, a robust framework for recognizing binding between T-cell receptors and antigens. Ideker et al. [21] employed meta-learning to train a versatile neural network model that can be easily adapted with minimal additional samples. Recent advancements in deep learning technologies have furthered the field of protein function prediction. Notably, DeepGOPlus and ProteinBERT have improved predictive accuracy by using convolutional NNs and transformer-based architectures to analyze protein sequences [22, 23] (The comparison result with DeepGoPlus is shown in Table S4–S8). Additionally, approaches like Graph2GO and TALE-cmap have utilized graph-based NNs and integrated protein structure data, respectively, to enhance function prediction [24, 25]. These methods underscore the significant contributions of deep learning in tackling complex PFP challenges. The literature mentioned above demonstrates the versatility and fast adaptation of meta-learning in training neural network models for few-shot biomedical tasks. Despite advances in meta-learning and PLMs, there remains a gap in the efficient integration of these techniques to address the unique challenges of protein function prediction. Current methods often require extensive retraining or adaptation phases, emphasizing the need for a more integrated approach that can leverage the strengths of both meta-learning and PLMs to deliver robust results across multiple protein function prediction tasks.

Here, we propose a pilot study, DeepPFP, based on Model-Agnostic Meta-Learning (MAML) [17] and ESM2, aiming to learn universal protein-function mapping relationships for rapid adaptation in few-shot tasks. In the first stage, protein sequences are represented using the ESM2, which is based on the BERT architecture [26]. ESM learns the interaction patterns between pairs of amino acids via an attention mechanism and captures the functional effects of sequence variation. We designed a simple multilayer neural network trained via meta-learning as a decoder to perform linear mapping of ESM representations to functions, and to learn generalized patterns across different datasets for rapid adaptation in the few-shot tasks. In the second stage, we performed finetuning on a new task, demonstrating its ability to rapidly adapt to few-shot tasks by gradually reducing the data volume. We conducted experiments on six DMS datasets, including five previously used by Sam et al. [6] (details can be found in the Method section), as well as a SARS-CoV-2 dataset for external validation. To increase the reliability of our experiments and evaluate the effectiveness of using PLMs to represent amino acid sequences, we compared their prediction ability with convolutional neural networks (CNNs) on multiple datasets for a single task. The results showed that PLMs improved the Pearson’s correlation coefficient (PCC) for the regression task by an average of 6.570%. Due to model degradation in CNNs, the improvement reached 13.569% on the Ube4b data. We then used a leave-one-out cross-validation approach where each of the five datasets was removed as a test set. After pre-training with meta-learning on the remaining four datasets, our model was able to converge quickly on the test set, resulting in an average PCC improvement of 0.622%. Based on these findings, we reduced the size of each dataset, and in most cases, the MAML-based decoder resulted in a significant improvement. Finally, we performed external validation of our method using the SARS-CoV-2 receptor-binding domain (RBD) amino acid mutations DMS dataset from Starr et al [27]. Despite the Uniform Manifold Approximation and Projection (UMAP) [28] visualization indicating the ESM’s representation was ineffective, DeepPFP still improved prediction performance. Applying this approach to multiple independent repeat experiments, we found that it reduced the data dependency of NNs and improved prediction performance on few-shot tasks.

## Result

### Overview of DeepPFP

In this study, we proposed a multi-staged AI framework DeepPFP for modeling the sequence-function relationship. DeepPFP is an encoder–decoder architecture trained using the MAML framework. Unlike previous sequence-function mapping methods, which may not generalize well beyond a limited dataset, DeepPFP can rapidly adapt to new tasks by learning from out-of-domain datasets.

### Model structure

**Figure 1:**
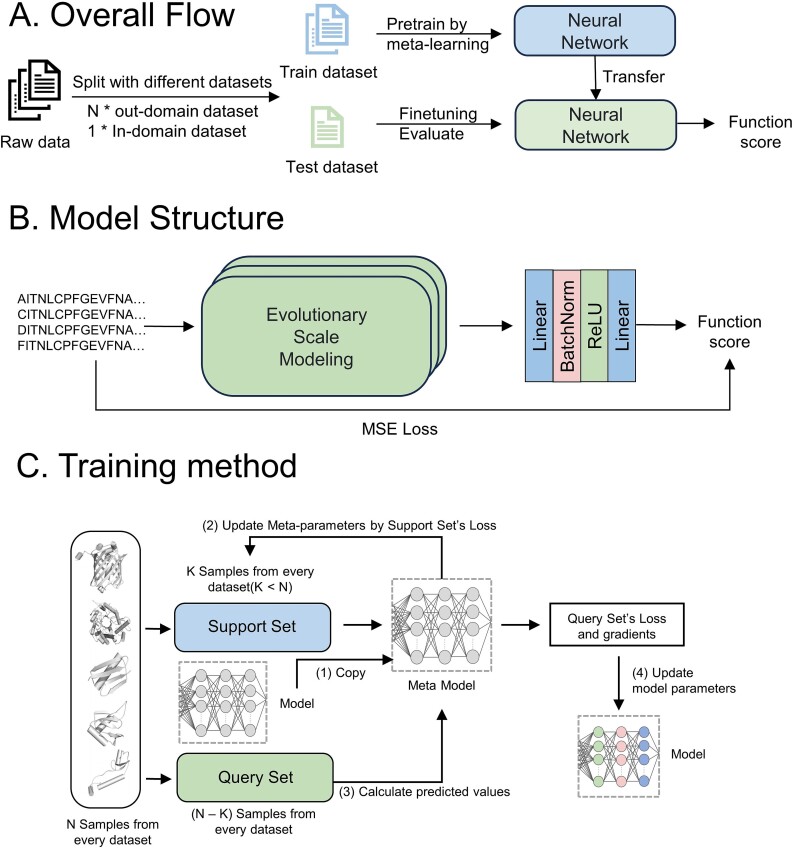
Study design. (A) the overall flow of DeepPFP. We use the out-domain dataset to improve the prediction performance of the few-shot task (in-domain dataset). (B) Model structure. (C) Training method of MAML. We use support set to update the Meta-parameters, then calculate the loss by the updated Metamodel. In the outer loop, we update the original model parameters using the above loss.

As shown in Fig. 1B, we utilized ESM as the encoder of DeepPFP, enabling the model to understand how amino acid substitutions affect various functions. ESM is a pre-trained PLM based on a Transformer Encoder and is trained using Masked Language Modeling (MLM). To verify whether PLMs can efficiently extract sequence-function mappings, we employed UMAP to visualize the individual datasets, coloring them according to label values. The results demonstrate that ESM accurately reflects the impact of variation on functional scores. The decoder consists of simple linear layers, batch norm layers (BN), and ReLU activation functions. The output layer of the decoder directly receives the hidden vector as input and outputs the functional score for each sequence.

### Training architecture (MAML)

The goal of meta-learning is to derive parameters that can be rapidly adapted to new tasks. The entire training architecture is based on MAML, a model-agnostic and user-friendly framework. Through training with representation vectors from various similar datasets via MAML, the model develops the capacity to analyze and reason about the effects of distinct site mutations on sequence-function relationships. It predicts functional scores and achieves excellent generalization across various datasets.

### Predictor

The trained network can be fine-tuned on a few-shot dataset to predict the function of protein variants not present in the training set. We found that pre-trained models using MAML achieved rapid convergence of losses on new datasets. To evaluate the performance and reliability, we conducted external validation using DMS data of SARS-CoV-2.

### PLM-based function prediction model compared to CNN

Numerous studies have validated the effectiveness of CNNs in similar tasks within computational biology, demonstrating superior performance in recognizing subtle yet critical patterns in sequence data that correlate with specific protein functions. But PLMs, such as BERT and its variants, have transformed the field of natural language processing and are increasingly being adapted for bioinformatics due to their ability to capture contextual relationships within data. To avoid the influence of different deep learning frameworks (TensorFlow and PyTorch), data division methods, and other factors, we replicated the CNNs model that outperformed all others in the Gelman’s study [6]. Ten-fold cross-validation on several supervised function prediction tasks utilized the same data sampling method to compare the CNN with the PLM. We collected the final accuracy scores from the ten-fold cross-validation and generated box plots to visually represent the data.

**Figure 2:**
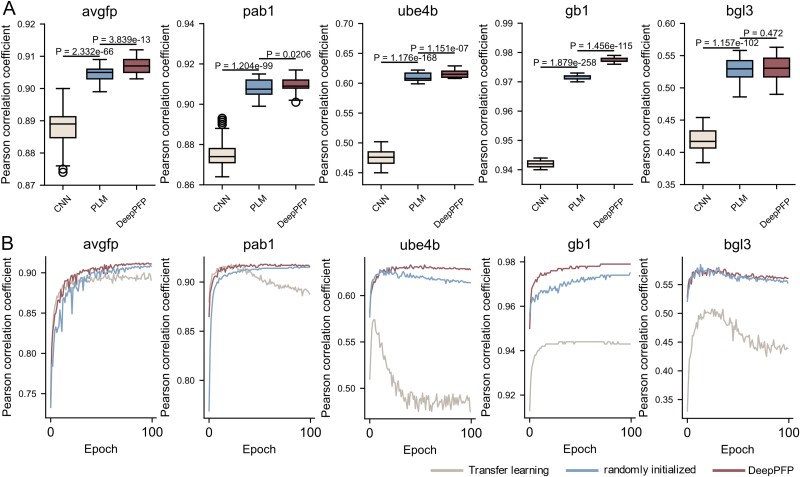
The performance of CNN, PLM, and DeepPFP. When using all the data, PLM performs better than CNN, and DeepPFP (PLM-MAML) further improves the prediction accuracy. (A) the results of multiple independent repeat experiments. (B) the training PCC curves for the first fold on every dataset. CNNs suffer from network degradation.

As shown in Fig. 2A, the results indicated that PLMs consistently outperformed CNNs. In experiments on all five datasets with a split rate of 0.8, the average PCC scores for CNN on the test sets were 88.807%, 41.940%, 94.203%, 97.508%, and 47.474%. In contrast, PLMs achieved PCC scores of 90.453%, 52.890%, 97.189%, 90.809%, and 61.043%. Compared to CNN, PLMs exhibited increases in PCC across five datasets as follows: 1.645%, 10.950%, 3.227%, 3.441%, and 13.569%, respectively.

This was surprising because CNN is a supervised learning method that relies on a specific dataset. Gradients are directed in the appropriate direction to optimize and capture the impact of different mutations on sequence-function mapping. However, the PLMs’ encoder does not participate in the backward process. This indicates that PLMs capture the functional landscape of the protein, learn complex protein functional information, and can be easily applied to new tasks without specialized adaptation.

During the experiment, we observed that CNN exhibited a phenomenon of network degradation across multiple datasets, characterized by an abrupt increase in training loss and a sharp decline in PCC. We have plotted the training PCC curves for the first fold, as shown in Fig. 2B. On the Ube4b dataset, where the network degradation was most severe, the highest PCC was 0.575; however, as training progressed, the PCC dropped by 0.101, resulting in a final result of only 0.474. On datasets without network degradation in CNN, there was an improvement of more than 1%.

### MAML-based training method compared to end-to-end training

We aimed to use out-domain data to enhance the performance of an in-domain dataset and trained DeepPFP (a rapidly adaptive model using MAML) on the five datasets above, thereby improving its predictive capabilities for new tasks. We conducted a leave-one-out analysis by separately removing each of the five public DMS datasets during training and subsequently using it as the test task. The method differs from traditional K-fold cross-validation in that each part is not randomly sampled from the same dataset, but rather from out-domain datasets. This resulted in the training set and test set having different distributions. Because multiple datasets were to be trained simultaneously, we standardized each dataset separately (see Method section).

The performance of DeepPFP was evaluated on five datasets using a randomly initialized decoder as a benchmark. Generally, across all experimental sets, the pre-trained DeepPFP demonstrated superiority over the randomly initialized model. It’s worth noting that while MAML is particularly suited for few-shot learning, the size of these sequence-function datasets exceeds 26 653.

To elaborate, using PCC as the metric for evaluation, the results for DeepPFP were 90.716%, 53.092%, 97.737%, 90.949%, and 61.562%, which improved by 0.263%, 0.202%, 0.409%, 0.140%, and 0.519% on each of the five datasets, respectively.

The performance within each dataset deserves further analysis. For instance, datasets Ube4b and Bgl3 still exhibited slight network degradation when using PLMs as an encoder. Specifically, dataset Bgl3, even when utilizing DeepPFP, continued to demonstrate the phenomenon. Compared to the epoch with the best results, the PCC in the final stage decreased by ~2%.

### Down-sampling test: MAML-based can effectively reduce training data size

The quantity of data was the primary factor that influenced the performance of few-shot tasks differently. Larger datasets enabled the randomly initialized decoder to receive sufficient training, thereby bringing its PCC close to that of DeepPFP. Conversely, the smaller data volumes led to significant fluctuations in predictions, increasing the uncertainty of results and complicating direct comparisons between the two methods.

To mitigate the above uncertainties and comprehensively evaluate the enhancement provided by MAML for the few-shot task, we conducted five independent repeat experiments on all datasets. We used sample sizes of [500, 600, 700, 800, 900, 1000, 2000, 3000, 4000, 5000] from each dataset for 10-fold cross-validation. The down-sampling was performed using a stratified approach to maintain a representative distribution of the different classes of protein functions. This method ensured that despite the reduction in data volume, the diversity and complexity of the dataset characteristics were preserved, allowing us to evaluate the model’s performance accurately across different scenarios.

For varying data volumes, we designed three sets of experiments: a transfer-learning-based model, a randomly initialized model, and DeepPFP (MAML-based model), respectively.

**Figure 3:**
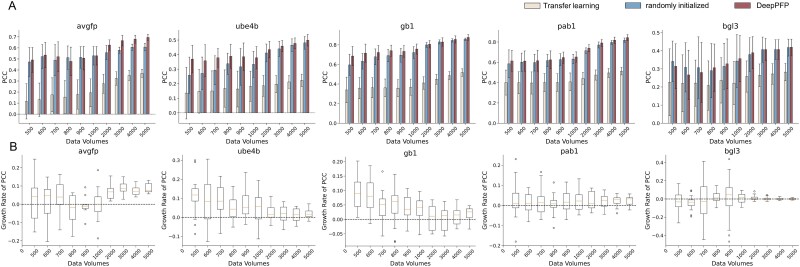
The influence of various size of support sets on performance of our pilot study. (A) we compared the performance of traditional transfer learning, random initialization, and DeepPFP. Traditional transfer learning suffers from the domain shift problem and produces poor results. Our method can improve the prediction results, especially when the data volume is reduced. (B) To demonstrate the advantages of DeepPFP more clearly, we calculated the improvement of DeepPFP on the results. The pattern shown in fig. A is again depicted. When reducing the data volume, the improvement is large, but it may be unstable.

As shown in Fig. 3A, we showed the experimental results on five public datasets, where the horizontal axis represents the data volumes and the vertical axis represents the PCCs. In each sub-figure, the beige color represents transfer learning, the blue color represents the randomly initialized model, and the red color represents DeepPFP. Due to the domain drift phenomenon, the PCC for transfer learning decreases. Notably, for the datasets avGFP and Ube4b, the errors are significant, with some results even demonstrating negative correlations (avGFP-500, avGFP-600, Ube4b-500). In contrast, DeepPFP consistently outperforms other models across all datasets, with its lowest values still surpassing the average performance of the randomly initialized model (e.g., avGFP-3000, avGFP-4000, avGFP-5000, Ube4b-500, gb1–500, gb1–600). However, MAML performs poorly on the Bgl3 dataset. This poor performance is inferred to be because the remaining datasets are less similar to it in terms of statistical data distribution and UMAP downscaling.

To better illustrate the improvement of DeepPFP in prediction results, we calculated the relative percentage of PCC improvement using the following formula: 


$$ pcc\_ increase=\frac{\left( pcc\_ meta- pcc\right)\ }{pcc} $$

We use box plots to visualize the percentage lift. As shown in Fig. 3B, when data volumes are small, the error range is larger and there are more outliers; however, as the data volume increases, the boosted results tend to stabilize. Consequently, while the error decreases, the improvement ratio also diminishes.

Moreover, as data volumes increase, the prediction results for all methods improve, substantiating that the outcomes of deep learning are intrinsically linked to the quantity of data.

### Case study: Validation of PLM-MAML with S-ACE2 affinity DMS data of SARS-CoV-2

SARS-CoV-2 enters human cells via the spike (S) protein, which specifically binds to the ACE2 receptor on host cells. This S protein is primed through proteolytic cleavage at the S1-S2 boundary by the host enzyme furin, activating it for interaction with ACE2. The S1 subunit contains a RBD that directly engages with ACE2. Upon this engagement, the S protein undergoes a significant conformation change, revealing the fusion peptide within the S2 subunit. This peptide embeds itself into the host cell membrane, facilitating the merging of viral and cellular membranes, which is a critical step for viral entry. This fusion allows the viral genome to enter the host cell, initiating infection. Therefore, understanding and predicting the binding affinity between RBD of the S protein and ACE2 antibodies is crucial for developing therapeutic strategies and vaccines against COVID-19.

And to further evaluate DeepPFP’s generalizability to new datasets that were not part of the initial training process, we conducted tests using the DMS dataset of the SARS-CoV-2 Spike protein RBD. Initially, the data were downscaled using UMAP and visualized. The observation revealed challenges in differentiating points of varying colors, indicating the difficulty of ESM in effectively downscaling the SARS-CoV-2 sequence vector directly. The model was then pre-trained using MAML on the five datasets. We compared the effects of a DeepPFP with a randomly initialized model on the results across different data volumes. The improvement of DeepPFP over the randomly initialized model was not stable when the data volumes were small. However, as data volumes increased, the results of the DeepPFP significantly surpassed those of the randomly initialized model. It can be inferred that for the SARS-CoV-2 protein, while MAML had limited feature extraction capabilities, MAML utilized knowledge gained from out-of-domain data to improve prediction accuracy.

## Discussion

Sequence-function mapping is highly complex, involving thousands of molecular interactions, which can profoundly affect the development of protein-related fields. For example, protein expression or catalytic activity is determined by its amino acid sequence, while protein engineering focuses on finding the protein that performs a specified function. Using deep learning for sequence-function mapping and protein function prediction can effectively narrow the protein sequence space and reduce the cost of wet experiments. Drugs work by binding to their targets, and drug discovery is often conducted by predicting the affinity between proteins and their targets; thus, the function of the protein has a significant influence on this binding process.

In this study, we have demonstrated the usability and performance of DeepPFP through experimental validation. It achieves performance boosts across multiple datasets and adapts effectively to the few-shot tasks. Overall, this research represents one of the efforts to enhance the performance of sequence-function mapping. DeepPFP is computationally efficient and offers numerous advantages for sequence-function mapping prediction. It accomplished this by computing and storing representation vectors using ESM and enhancing generalizability through Meta-learning. Its scalability and rapid adaptation for unknown few-shot tasks make it suitable for researching unknown proteins or developing extrapolatable frameworks, potentially opening new avenues for exploring unanswered questions in biomedicine.

However, DeepPFP also possesses certain limitations that warrant further in-depth exploration.

### Directions for algorithmic improvement

There are two factors concerning the training of NNs. First, the computation of second-order partial derivatives consumes substantial computational resources and extends training time. Although first-order approximations exist to mitigate this problem, they tend to undermine the generalization of models. Furthermore, when training large end-to-end PLMs using meta-learning, gradient accumulation due to the summation of losses frequently leads to ‘out of memory’ errors. Baroni et al. [14] have demonstrated that NNs optimized for meta-learning by compositionality, can mimic human systematic generalization in a head-to-head comparison. Therefore, if the high computational overhead associated with meta-learning is alleviated, it becomes feasible to train networks capable of learning to address significant challenges in biomedicine. Second, optimizing the outer loop necessitates multiple backward passes through the inner loop. Computing each layer of the network multiple times could lead to gradient vanishing or gradient exploding. When gradients vary widely, parameter updates become unstable, resulting in more random and less robust outcomes. Researchers have made strides in addressing both issues; however, complete solutions remain elusive. I contend that resolving these challenges may require the development of more advanced theories.

### Correlation analysis between UMAP and results

To elaborate on the capacity of sequence-function mapping, we employed UMAP to reduce the dimensions of the embedding vector (Fig. 5D). In the latent space, variants are color-coded based on their functional scores; the darker the red, the higher the score, and the darker the blue, the lower the score. Across all datasets, variants with similar scores consistently clustered within nearby regions on Cartesian coordinates. Most notably, data with high scores, represented in red, typically clustered in the central region of the scatter plot. This was particularly evident in the datasets avGFP, Pab1, and Ube4b, where the blue regions surrounded the red regions—visualizing these relationships between sequences within the latent space illustrated how ESM understands protein sequences and learns the biological features embedded in sequences. The observed similarity across these datasets further confirmed ESM’s capability to generalize to capture the sequence–function mapping of the unknown protein. This offered an excellent foundation for employing MAML to easily adapt to new tasks. For dataset Bgl3, the projection of the function scores in Cartesian coordinates varied considerably from the other four datasets. Given that ESM’s results were already superior to those of CNNs, it would be incorrect to assume that ESM failed to learn the biological features related to function. Hence, we can conclude that the pattern of dataset Bgl3 differs from the rest of the datasets in the sequence-function mapping. This indicated that using MAML to learn the effect of similar mutations on function score changes on the other datasets was not feasible for Bgl3. For the dataset Ube4b, the PCC of DeepPFP was further improved, addressing the degradation problem. This precisely demonstrated that during the training process, MAML was able to capture a certain common pattern of sequence-function mapping and learn the biological significance of protein function. For other datasets, it was found that most of their blue points were in the periphery. Therefore, the performance of DeepPFP was similarly improved.

**Figure 4:**
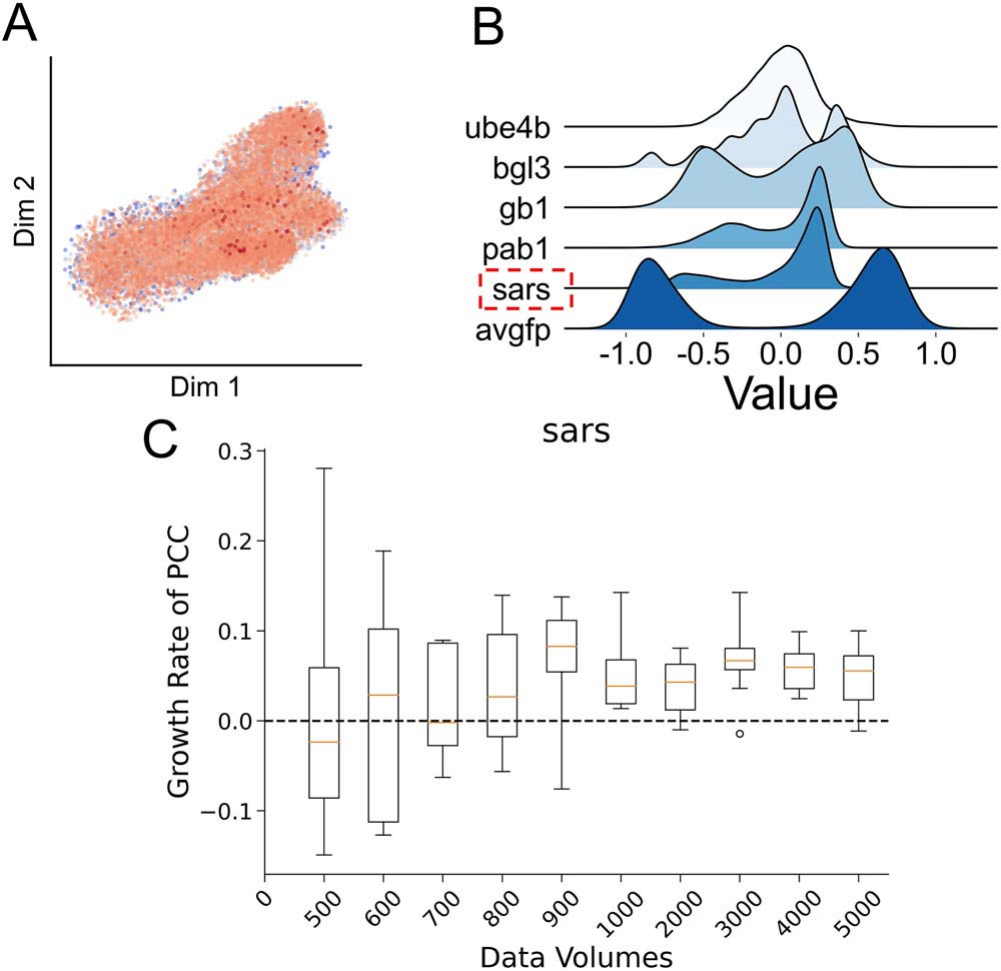
(A) UMAP visualization of the SARS-CoV-2 DMS dataset. (B) Data distribution visualization. Although the distribution of the UMAP plot has low similarity to the rest of the datasets, the distribution of its labels is like Pab1. This may have led to the possibility that MAML was able to improve predictive performance. (C) the improvement of DeepPFP.

**Figure 5:**
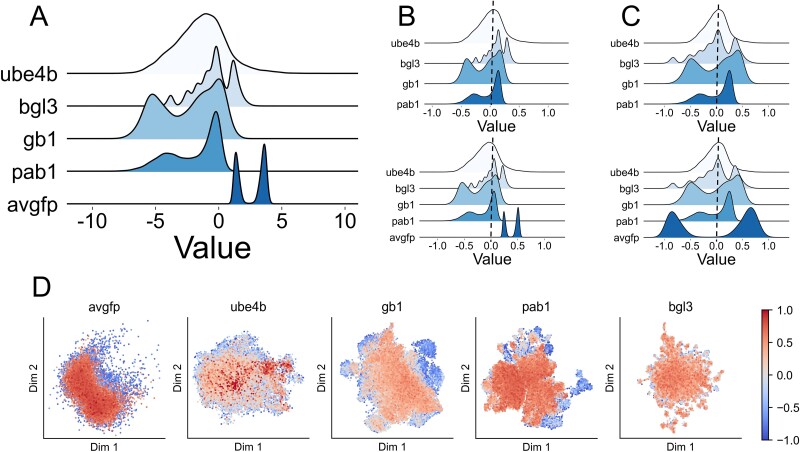
Data distribution and UMAP visualization analysis. (A) Joint-STD: The original distribution of labels. (B) Datasets are merged and then standardized. Introducing new datasets requires re-standardization of old datasets and introduces a bias to the old datasets. (C) IND-STD: Datasets are standardized separately. The introduction of a new dataset does not affect the old datasets and has less impact on the model. (D) UMAP visualization of DMS dataset. Using UMAP to reduce the dimensions of the embedding vector. In the latent space, variants are color-coded based on their functional scores; the darker the red, the higher the score, and the darker the blue, the lower the score.

### Biological implications of the model’s predictions

A key biological implication of our model’s predictions is its ability to discern meaningful relationships between protein sequence variations and their functional outcomes. UMAP visualizations of the latent space generated by DeepPFP show that variants with similar functional scores cluster together. This clustering demonstrates that the model effectively captures the biochemical and structural features underlying protein function. For example, in the SARS-CoV-2 RBD dataset, despite challenges in downscaling sequence vectors, DeepPFP successfully pinpointed mutations that significantly impact ACE2 receptor binding affinity. This is biologically significant, as changes in binding affinity can affect viral infectivity and transmissibility. Moreover, by accurately predicting specific amino acid substitutions’ effects, our model can identify critical residues vital for protein stability and activity. This information is invaluable for protein engineering, such as designing stable enzymes or developing vaccines and therapeutics targeting viral proteins. In the context of SARS-CoV-2, understanding mutations’ effects on spike protein-ACE2 interactions can guide the development of neutralizing antibodies and inhibitors.

### Future work

In our forthcoming research, we aim to develop more logical meta-learning-based models. This effort will involve collecting higher-quality data, analyzing protein data patterns, and delving deeper into strategies for addressing few-shot prediction tasks. Our objective is to enhance the performance of protein-function mapping prediction and protein–protein interaction prediction in the biomedical field.

## Method

To validate the fundamental few-shot sequence-function prediction method within this study, it is crucial to accumulate a dataset that reveals the complex correlations between protein mutant sequences and their functional interdependencies. Thus, we have initiated experimental efforts using datasets derived from deep mutation scanning. Comprehensive details will be furnished in the dedicated ‘Data’ section. We employed ESM to represent the experimental data, specifically employing the model esm2_t48_15B_UR50D, which comprises 15 billion parameters. Based on the obtained representation vectors, we utilized a linear layer with 5120 neurons to learn the relationship between representation vectors and functionality. To overcome the challenges of mapping sequences to functions in few-shot datasets, we adopted the third-party library learn2learn [29] to implement the MAML algorithm during the experimental design phase. Ultimately, we used PCC as the metric to assess the task and quantitatively evaluate the performance of various computation methods when applied to few-shot data tasks.

### Data and preprocessing

#### Data overview

DMS is a high-throughput experimental technique used to assess the effects of a wide range of genetic mutations, typically on the function or stability of a protein. This method combines site-directed mutagenesis, where nearly every possible amino acid substitution at each position in a protein is created, with a selection process to evaluate the functional impact of these mutations. For preprocessing steps of DMS datasets, without special preprocessing, only the mutation information is integrated into the original sequence to correspond to its label. For example, D120G in a dataset represents the 120th site changing from D to G.

There are six deep mutation scanning datasets publicly available, each corresponding to different proteins. Full sequences were mutated via deep mutation scanning, and fitness scores were calculated. These datasets vary in size and the lengths of the proteins. Detailed information on the data is provided in Table 1. This study evaluated the ability of two NNs to learn protein-function mapping, CNNs and ESM, respectively. The input for both approaches was a protein sequence file (.fasta). CNN can be trained directly using the end-to-end method, whereas ESM maps the protein sequences into a tensor of Pytorch, storing it as a floating array (.pt).

**Table 1:** Basic information of the DMS datasets.

	Description	Function	Length	Variants	Ref.
avGFP	Green fluorescent protein	Fluorescence	237	54,024	[30]
Bgl3	β-glucosidase	Hydrolysis of β-glucosidic linkages	501	26,653	[31]
GB1	Protein G B1 domain	IgG binding	56	536,084	[32]
Pab1	Pab1 RNA recognition motif (RRM) domain	Poly(A) binding	75	40,852	[33]
Ube4b	Ubiquitination factor E4B U-box domain	Ubiquitin-activating enzyme activity	102	98,297	[34]
RBD	SARS-CoV-2 spike receptor-binding domain	ACE2 binding	201	32,160	[27]

**Table 2:** Pseudo-code for model-agnostic meta-learning.

Model-Agnostic Meta-Learning	
Require: $p\left(\mathcal{T}\right)$: distribution over tasks	
Require: learning rates: $\alpha =0.01,\beta =0.005$	
loop iterations: $outer=1000, inner$ is determined by datasets	
1	Randomly initialize $\theta$
2	While not done do
3	Sample batches task ${\mathcal{T}}_i\sim p\left(\mathcal{T}\right)$
4	for all ${\mathcal{T}}^t$ do
5	Sample K datapoints $\mathcal{D}=\left\{{x}^{(j)},{y}^{(i)}\right\}\ from\ {\mathcal{T}}_i$
6	Evaluate ${\nabla}_{\theta }{\mathcal{L}}_{{\mathcal{T}}_i}\left({f}_{\theta}\right)$ using $\mathcal{D}$ and $\mathcal{L}$ in MSE
7	Compute adapted parameters with gradient descent: ${\theta}_i^{`}\leftarrow \theta -\alpha{\nabla}_{\theta }{\mathcal{L}}_{{\mathcal{T}}_i}\left({f}_{\theta}\right)$.
8	Sample datapoints ${\mathcal{D}}_i^{\prime }=\left\{{x}^{(j)},{y}^{(i)}\right\}$ for meta-update
9	End for
10	Update $\theta \leftarrow \theta -\beta{\nabla}_{\theta}\sum_{{\mathcal{T}}_i\sim p\left(\mathcal{T}\right)\ }{\mathcal{L}}_{{\mathcal{T}}_i}\left({f}_{\theta_i^{\prime }}\right)$using each ${\mathcal{D}}_i^{\prime }$ and ${\mathcal{L}}_{{\mathcal{T}}_i}$
11	End while

Especially the SARS-CoV-2 dataset, given the global impact of the COVID-19 pandemic, it is highly relevant for current research. It provides a unique opportunity to apply our model to a pressing real-world problem—understanding protein interactions involved in the virus’s pathogenesis. This dataset includes sequences of the SARS-CoV-2 Spike protein and its interactions with the human ACE2 receptor, critical for viral entry into host cells. Analyzing these interactions can directly contribute to the development of effective vaccines and therapeutic agents.

#### Z-score standardization

Data distribution is a key point of machine learning. The datasets mentioned above exhibit diverse distributions, and using MAML for mixed training can affect the model’s performance in sequence-function mapping. To enhance the performance, we applied Z-score normalization to scale our labels. Z-score normalization is a data scaling technique that standardizes label values by transforming them to have a mean of zero and a standard deviation of one.

This transformation is accomplished by subtracting the mean from each value and then dividing it by the standard deviation. The resulting values, commonly referred to as Z-scores, indicate how many standard deviations each data point is from the mean. The Z-Score normalization for one dataset can be expressed as follows:


$$ x=\frac{x-{x}_{mean}}{x_{std}} $$

${x}_{mean}$ represents the mean and ${x}_{std}$ represents the variance.

#### Multi datasets standardization method

DeepPFP is capable of fitting multiple datasets simultaneously, necessitating the selection of an appropriate standardization approach. We evaluated two methods for standardizing multiple datasets: individually standardizing each dataset (IND-STD) and standardizing all data combined (Joint-STD). To determine an appropriate data standardization method, the two approaches were separately visualized. Initially, the label distributions of Ube4b, Bgl3, GB1, and Pab1 were analyzed using both methods, followed by the inclusion of the avGFP data and re-analysis. IND-STD demonstrated several advantages. First, as illustrated in Fig. 5A, it maintains the original distribution of the data, scaling labels uniformly within the range of −1 to 1 (as shown in Fig. 5C). In contrast, the distribution after standardization using the other method is influenced by the label distribution in the original dataset, leading to increased bias (Fig. 5B). Second, the addition of new data does not require re-standardizing the existing dataset, and it does not affect the network’s ability to predict the fitted dataset. The focus in training the neural network is to capture the changes in function scores caused by mutations within a specific dataset, rather than modeling the relative relationships between different datasets. This is why IND-STD was chosen.

### Loss function and evaluation metrics

#### Mean-squared loss

We primarily focus on the regression problem of mapping sequences to functions, where MSE is commonly employed as the loss function for regression tasks. MSE quantifies the degree of alignment between the estimated values and the actual values by computing the mean squared error, and its definition is straightforward. If a batch contains n data points, the MSE for that batch can be expressed as follows:


$$ MSE=\frac{1}{n}\sum_{i=1}^{i=n}{\left({y}_i-{\hat{y}}_i\right)}^2=\frac{1}{n}\sum_{i=1}^{i=n}{\left({y}_i-f\left({x}_i\right)\right)}^2 $$

MSE values range from 0 to +∞. For a given batch of data, a lower MSE indicates a closer alignment between the predicted values and the ground truth. In deep learning, the MSE loss function features a smooth, continuous curve that is differentiable at all points. This characteristic is advantageous for its application in gradient descent algorithms, facilitating parameter updates. Additionally, as the error decreases, the gradient also reduces, enhancing the convergence rate of the loss.

#### Pearson correlation coefficient

We employ the PCC to quantify the correlation between predicted function scores and actual scores, providing a normalized measure of covariance. The computation of PCC yields values within the range of −1 to 1, reflecting the linear relationship between variables. A value of −1 signifies a complete negative correlation, 0 indicates no correlation, and 1 denotes a perfect positive correlation.


$$ Cov\left(x,y\right)=E\left[\left(x-\mu x\right)\left(y-\mu y\right)\right]\qquad\qquad\qquad\qquad\qquad\ \ \ \qquad $$

$$ pcc\left(x,y\right)=\frac{Cov\left(x,y\right)}{\sigma x\sigma y}=\frac{\sum_{i=1}^{i=n}\left({x}_i-{\mu}_{x_i}\right)\left({y}_i-{\mu}_{y_i}\right)}{\sqrt{\sum_{i=1}^{i=n}{\left({x}_i-{\mu}_{x_i}\right)}^2}\sqrt{\sum_{i=1}^{i=n}{\left({y}_i-{\mu}_{y_i}\right)}^2}} $$

### ESM

Instead of employing one-hot encoding, we utilize ESM to encode sequences. The one-hot expression matrix exhibits two notable shortcomings: its sparsity, and the independence of its variables, which lead to a semantic gap. Encoding amino acids using parameter-learning embedding allows the bases to be expressed as low-dimensional, dense, computable vectors. Subsequently, the Transformer Encoder is applied to learn the interactions between different bases and the deep, abstract biochemical features expressed by the sequence. Moreover, using a uniform model to encode different datasets ensures consistent vector lengths, eliminating the necessity to manage varying vector lengths and facilitating the use of a generalized network for experiments. ESMFOLD is adept at understanding the structural, functional, and biochemical properties of protein sequences. As the parameter size and dataset increase, the model emerges with more powerful capabilities (Comparison of different ESM2 models in Fig. S2). We chose esm2_t48_15B_UR50D as the encoder, which consists of 48 layers and has an Embedding Dim of 5120, resulting in a staggering parameter count of up to 15 billion. The model was trained by Facebook researchers using the MLM method on the UR50/D 2021_04 dataset.

### Uniform manifold approximation and projection

The ESM outputs a latent representation of the sequence, with the hyperparameter ‘—include’ being set to ‘mean’. As a result, each variant yields a 5120-dimensional vector. To facilitate visualization while maintaining spatial relationships between variants, we employed UMAP to project the latent representation into 2D space. The Python package ‘umap-learn’ was utilized for computing this projection. The 2D visualization illustrates how the ESM understands variants. By color-coding each variant according to its functional score, we demonstrate the ESM’s capacity to discern and represent the nuances of the variant.

### Running parameters of our framework

To learn the linear relationship between the representation vector and score, we developed a simple neural network model. In the input layer, we implemented parameter $reduction$ for dimensionality reduction of the representation vector. Assuming the initial dimension of the input vector is $p$. This dimension is reduced to $p/ reduction$ following the input layer. The hidden layer, maintaining this dimensionality, ensures that both its input and output dimensions match, aligning with the output of the input layer. The purpose of this layer is to avoid a neural network with only input and output layers having too few parameters to fit the representation vector-function mapping. Following the input and hidden layers, we employ a BN to normalize the vectors. The ReLU activation function is subsequently applied, which sets all negative feature values to zero. The functional expression for ReLU is $f=\mathit{\max}\left(0,x\right)$. This action makes the neurons sparser, which can improve computational efficiency, while retaining the positive values that are useful for the results, allowing the model to better explore the relationship between the representation vectors-scores. Finally, the output layer maps the vectors to the score without going through BN and ReLU, thus retaining the output values. The parameter settings and hardware configuration of this framework can be seen in Table S1–S3 and Fig. S1.

### Model-agnostic meta-learning

Before adopting MAML, we tested various machine learning and gradient propagation methods, and the results were not as good as the current framework, as shown in Fig. S3–S8. Initially, the dataset for MAML is divided into two sets: a support set and a query set. The MAML architecture utilizes a two-loop structure, where the inner and outer loops have distinct learning rates, denoted by $\alpha$ and $\beta$, respectively. The inner loop copies the original model parameters $\theta$ and updates the parameters of the copied model within the loop using the support set. The loss is calculated based on the support set, and the gradient (${\nabla}_{\theta }{\mathcal{L}}_{{\mathcal{T}}_i}\left({f}_{\theta}\right)$) is derived. The model parameters for the $i\_ th$ batch are updated to ${\theta}_i^{`}$ by the equation: ${\theta}_i^{`}\leftarrow \theta -\alpha \ast{\nabla}_{\theta }{\mathcal{L}}_{{\mathcal{T}}_i}\left({f}_{\theta}\right)$. This would only update the copied model in the inner loop, independent of the original model. But within the inner loop, we would compute and record the $loss$ of the query set on the temporary model (copied model), and this recording would cause the accumulation of gradients. Subsequently, in the outer loop, the original model is updated based on the aggregated $losses$ from all the batches. To further explain, after finishing an inner loop, we get the aggregated $losses$ and update the original model parameters by the equation: $\theta \leftarrow \theta -\beta \ast{\nabla}_{\theta }{\sum}_{{\mathcal{T}}_i\sim p\left(\mathcal{T}\right)}{\mathcal{L}}_{{\mathcal{T}}_i}\left({f}_{\theta_i^{\prime }}\right)$. This update is dependent on the gradient of the inner loop; therefore, it is called “gradient by gradient”.

**Key Points**

- The novel framework, DeepPFP, was designed to rapidly adapt to out-domain sequence-function prediction tasks using ESM2 and MAML.
- In experimental validation, ESM2 combined with a simple decoder enhanced the PCC for the regression task by an average of 6.57%.
- Notably, training on a subset of the Ube4b dataset with 500 samples led to a significant improvement in PCC, increasing by 0.11.
- Effectively addressed the domain shift challenges in transfer learning within the field of biomedical data.

## Supplementary Material

deeppfp_sup_bbae579

## Data Availability

All datasets have been organized into FASTA files and uploaded to GitHub: https://github.com/deconvolution-w/DeepPFP/tree/master/data.

## Code availability

A cleaned version of DeepPFP’s code is freely available on GitHub: https://github.com/deconvolution-w/DeepPFP.
