# Supplementary material for: DeepPFP: a multi-task-aware architecture for protein function prediction
Source: Brief Bioinform. 2025 Feb 5;26(1):bbae579. doi: 10.1093/bib/bbae579 (PMC11794456; doi:10.1093/bib/bbae579)
Supplement: deeppfp_sup_bbae579 [file deeppfp_sup_bbae579.pdf]

DeepPFP: A Multi-Task-Aware architecture for protein function prediction
supplementary

Detailed technical specifications

Table 1. training conditions

parameter	values
batch size	32
lr	0.005
maml lr	0.01
iterations	1000
fas	5
split rate	0.8
optimizer	Adam
scheduler	StepLR

Table 2. hardwares requirements

hardware	type	numbers
CPU	Xeon(R) Gold 5320 CPU @ 2.20GHz	2
GPU	A6000(48G)	4
RAM	32	8

In the ESM2 inference stage, we recommend using A100, a GPU with 80GB of memory, or using A40 or A6000 as a replacement, but it needs to be modified from the source code to make its calculation accuracy half precision (i.e. use tensor.half() and model.half()).

Generalization

Early stopping

To enhance the generalizability and computational efficiency of our models, we implemented an early stopping technique. Early stopping is a form of regularization used to avoid overfitting during the training phase of machine learning models. It works by monitoring the model's performance on a validation dataset at each epoch. If the model's performance on the validation set does not improve for a predefined number of consecutive epochs, known as the "patience" period, the training process is halted. This approach not only saves computational resources but also ensures that the model does not continue to learn idiosyncrasies of the training data that are unrepresentative of the general population, thereby improving the model's predictive performance on new, unseen data.

Table 3. Early stopping's parameter

parameter	value	notes
patience	10	Wait time after last validation
verbose	False	Prints a message for each validation
delta	0	Minimum change in the monitored quantity

Dropout Strategies

In our study, we did not employ dropout within the decoder because the ESM's pre-training already incorporates two specific dropout strategies to enhance model robustness and generalization. These are:

Multi-Head Attention's Dropout During training, it randomly deactivates a subset of neurons, which helps the network learn more robust features that aren't dependent on a few neurons. This increases model generalization by simulating the training of multiple networks. At test time, dropout is disabled, and the weights are adjusted to compensate for the dropout rate used during training.

Dropout Token During pre-training, specific amino acid residues in protein sequences are randomly masked with a <mask>token. The model then predicts these masked tokens based solely on the context provided by the surrounding residues, which strengthens its ability to infer structural and functional attributes of proteins from partial information.

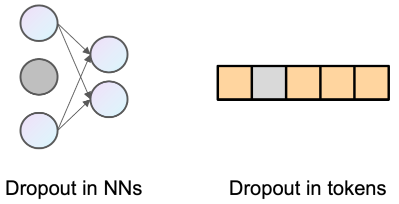

Fig. 1. Dropout Strategies

Pre work

Comparison of different Pre-trailed Models

For clarity, we compared the impact of different ESM models on the representation results. First, six ESM2 models with different parameters were used to represent the randomly sampled data, followed by dimensionality reduction using UMAP and clustering with K-Means. The silhouette score was used as the evaluation indicator for the clustering results to analyze the impact of different models. The experimental results indicated that the model with the largest number of parameters, esm2_t48_15B, achieved the best representation effect.

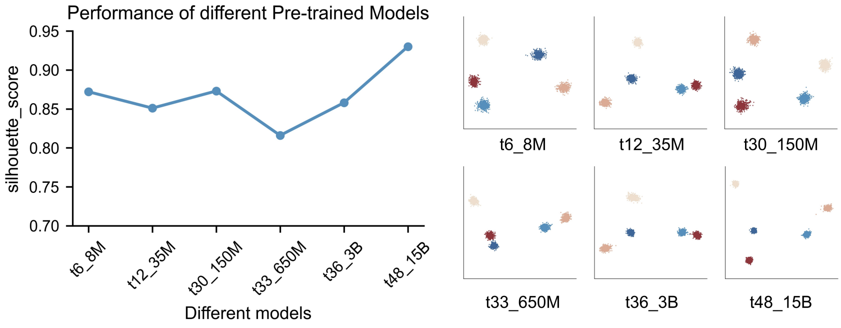

Fig. 2. Performance of different Pre-trailed Models. The left figure shows the clustering indicators for different model tables. The right figure is a two-dimensional scatter plot of different models after dimensionality reduction

Training methods

MAML

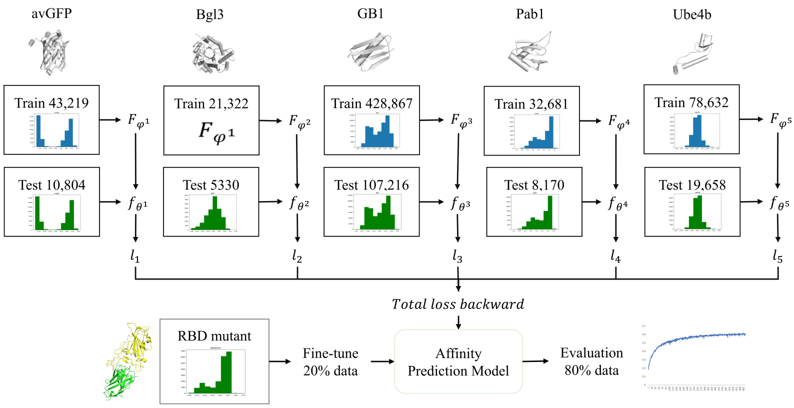

Fig. 3. Diagram of MAML.

single-loss

Unlike the previous algorithm, after calculating the loss of one batch for each set of data, backpropagation is immediately performed. The backpropagation process of each set of data will alternate.

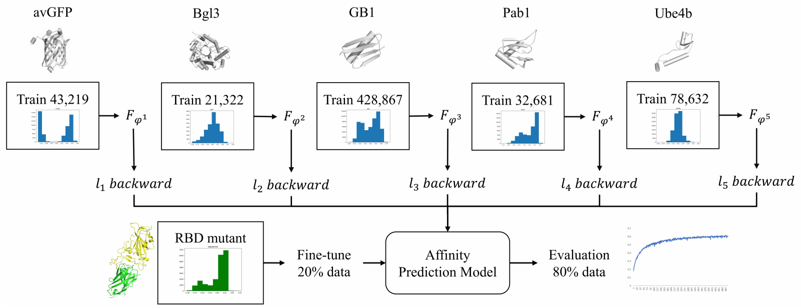

Fig. 4. Diagram of add-loss.

Add-loss

Similarly, all data was normalized before training. The idea behind this training method is inspired by MAML, where we calculate losses for each dataset separately and then add up all losses. We have created separate dataloaders for each group of data and calculated the loss separately during the calculation.

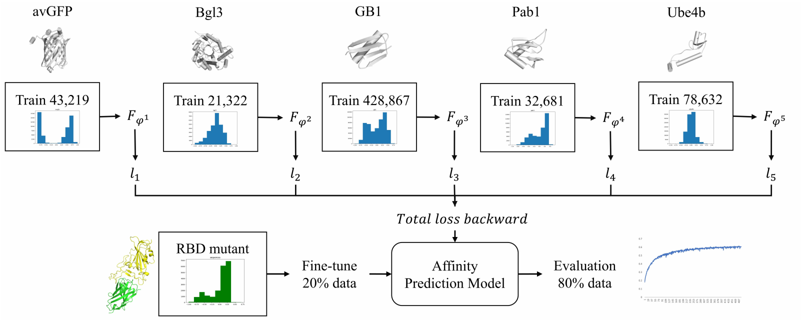

Fig. 5. Diagram of add-loss.

Mix-dataset

The mix-dataset method is to place all data in the same dataset and then perform random resampling. When we train, there are different amino acid sequence representation vectors in each batch of data, which have been mapped to the same dimension through ESM2, and training can be carried out without the need to learn from each other's strengths and weaknesses.

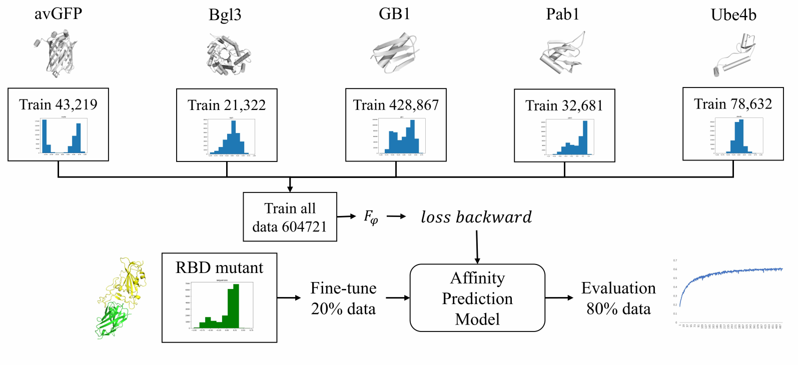

Fig. 6. Diagram of Mis-dataset.

Testing MSE Loss

We recorded the loss variation curve during the training process and found that after pre training with different training methods, MAML can quickly converge on the new dataset.

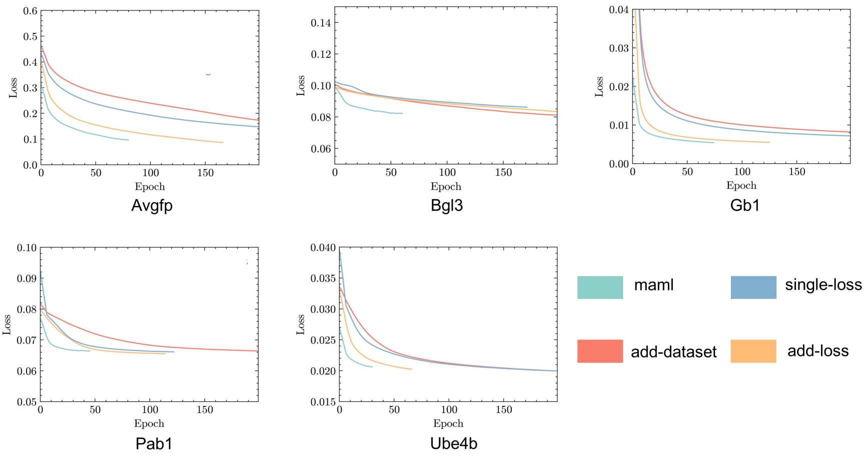

Fig. 7. Loss variation curve

Machine Learning

The performance of some machine learning methods on the dataset designed in this study.

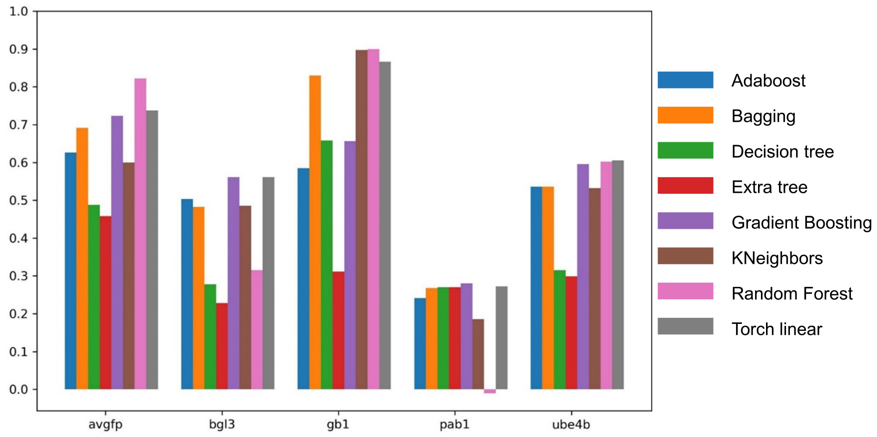

Fig. 8. The results of different machine learning methods

Architecture of CNNs

We replicated the best performing model in reference[1], which has different parameters across different datasets.

- avgfp
  conv1/kernel:0 Shape: [3, 40, 1, 128]
  conv1/bias:0 Shape: [128]
  conv2/kernel:0 Shape: [3, 1, 128, 128]
  conv2/bias:0 Shape: [128]
  conv3/kernel:0 Shape: [3, 1, 128, 128]
  conv3/bias:0 Shape: [128]
  conv4/kernel:0 Shape: [3, 1, 128, 128]
  conv4/bias:0 Shape: [128]
  conv5/kernel:0 Shape: [3, 1, 128, 128]
  conv5/bias:0 Shape: [128]
  dense1/kernel:0 Shape: [29056, 100]
  dense1/bias:0 Shape: [100]
  output/kernel:0 Shape: [100, 1]
  output/bias:0 Shape: [1]
  number of trainable parameters: 3,118,409

- bgl3
  conv1/kernel:0 Shape: [17, 40, 1, 32]
  conv1/bias:0 Shape: [32]
  dense1/kernel:0 Shape: [15520, 100]
  dense1/bias:0 Shape: [100]
  output/kernel:0 Shape: [100, 1]

output/bias:0 Shape: [1]
number of trainable parameters: 1,573,993

- gb1
  conv1/kernel:0 Shape: [17, 40, 1, 128]
  conv1/bias:0 Shape: [128]
  conv2/kernel:0 Shape: [17, 1, 128, 128]
  conv2/bias:0 Shape: [128]
  conv3/kernel:0 Shape: [17, 1, 128, 128]
  conv3/bias:0 Shape: [128]
  dense1/kernel:0 Shape: [1024, 100]
  dense1/bias:0 Shape: [100]
  output/kernel:0 Shape: [100, 1]
  output/bias:0 Shape: [1]
  number of trainable parameters: 747,081

- pab1
  conv1/kernel:0 Shape: [17, 40, 1, 128]
  conv1/bias:0 Shape: [128]
  conv2/kernel:0 Shape: [17, 1, 128, 128]
  conv2/bias:0 Shape: [128]
  conv3/kernel:0 Shape: [17, 1, 128, 128]
  conv3/bias:0 Shape: [128]
  dense1/kernel:0 Shape: [3456, 100]
  dense1/bias:0 Shape: [100]
  output/kernel:0 Shape: [100, 1]
  output/bias:0 Shape: [1]
  number of trainable parameters: 990,281

- ube4b
  conv1/kernel:0 Shape: [3, 40, 1, 128]
  conv1/bias:0 Shape: [128]
  conv2/kernel:0 Shape: [3, 1, 128, 128]
  conv2/bias:0 Shape: [128]
  conv3/kernel:0 Shape: [3, 1, 128, 128]
  conv3/bias:0 Shape: [128]
  conv4/kernel:0 Shape: [3, 1, 128, 128]
  conv4/bias:0 Shape: [128]
  conv5/kernel:0 Shape: [3, 1, 128, 128]
  conv5/bias:0 Shape: [128]
  dense1/kernel:0 Shape: [11776, 100]
  dense1/bias:0 Shape: [100]
  output/kernel:0 Shape: [100, 1]
  output/bias:0 Shape: [1]
  number of trainable parameters: 1,390,409

Other methods

We have replicated experiments using DeepGOPlus under identical conditions to provide a direct comparison of performance metrics across different data volumes. These tables show the Pearson Correlation Coefficient (PCC) scores for each dataset, highlighting areas where DeepPFP outperforms or underperforms in comparison to DeepGOPlus.

Table 4. performance of avgfp

data volums	DeepGOPlus	DeepPFP
500	**0.5669**	0.4904
600	**0.5355**	0.5352
700	**0.5974**	0.5200
800	**0.6760**	0.4778
900	**0.6540**	0.5067
900	**0.6980**	0.5285

Table 5. performance of ube4b

data volums	DeepGOPlus	DeepPFP
500	0.3003	**0.3688**
600	0.1664	**0.3573**
700	0.3272	**0.3780**
800	0.3465	**0.3884**
900	0.3094	**0.3854**
1000	0.2668	**0.3949**

Table 6. performance of gb1

data volums	DeepGOPlus	DeepPFP
500	0.6401	**0.6850**
600	0.6499	**0.7036**
700	**0.7411**	0.7215
800	0.7240	**0.7394**
900	**0.7366**	0.7230
1000	**0.7656**	0.7387

Table 7. performance of pab1

data volums	DeepGOPlus	DeepPFP
500	0.1898	**0.6130**
600	0.1451	**0.6118**
700	0.1093	**0.6158**
800	-	**0.6239**
900	0.1657	**0.6461**
1000	0.1076	**0.6519**

Table 8. performance of bgl3

data volums	DeepGOPlus	DeepPFP
500	0.0623	**0.3144**
600	0.2031	**0.2664**
700	0.2100	**0.2801**
800	0.1735	**0.3064**
900	0.1620	**0.3320**
1000	0.1901	**0.3564**
